# PAS-based analysis of natural gas samples

**DOI:** 10.3389/fchem.2023.1328882

**Published:** 2023-12-21

**Authors:** Marc-Simon Bahr, Marcus Wolff

**Affiliations:** ^1^ Heinrich Blasius Institute of Physical Technologies, Hamburg University of Applied Sciences, Hamburg, Germany; ^2^ School of Computing, Engineering and Physical Sciences, University of the West of Scotland, Paisley, United Kingdom

**Keywords:** photoacoustic spectroscopy, main alkanes, interband cascade laser, natural gas, hydrocarbons

## Abstract

Photoacoustic spectroscopy (PAS) is well known for the detection of short-chain hydrocarbons, such as methane, ethane and propane, in the ppm (parts per million) or ppb (parts per billion) range. However, in the production process of natural gas and its combustion in gas-fired devices the composition, especially the concentrations of the main alkanes, plays a decisive role. Gas chromatography (GC) is considered the gold standard for natural gas analysis. We present a method to analyze natural gas samples by PAS. Furthermore, we describe a method to prepare storage gas samples, which are usually under atmospheric pressure, for PAS analysis. All measurements are validated by means of GC. The investigation allows conclusions to be drawn to what extent PAS is suitable for the investigation of natural gas samples.

## 1 Introduction

Natural gas is one of the most important energy sources of our time and plays a significant role in the global energy supply. It consists of 75%–99% methane, 1%–15% ethane and 1%–10% propane as well as other small amounts of longer-chain hydrocarbons such as butane and pentane, but also of small amounts of nitrogen, carbon dioxide and noble gases (Hammer et al., 2003). The exact composition of natural gas is strongly dependent on its origin (Decourt et al., 2014).

When drilling for gas production, gas samples are taken at various drilling depths. These samples have a composition similar to commercial natural gas and, based on the compositions of the samples, allow geological conclusions regarding the surrounding rock but also about the expected gas deposit (Wiersberg and Erzinger, 2007).

Furthermore, the knowledge of composition of natural gas is extremely important for an effective combustion. The proportions of the individual hydrocarbons determine the energy content of the natural gas and thus its calorific value. In a mixture the different components contribute according to their concentrations (Leicher et al., 2017; Park et al., 2021).

Optimal operating parameters of gas-fired devices depend on the composition because methane, ethane and propane require different amounts of oxygen for a complete (stoichiometric) combustion (Demoulin et al., 2008). If the ratio of oxygen to gas components is not correct, incomplete combustion can occur, reducing energy efficiency and potentially causing harmful emissions. The knowledge of the composition helps to set the right combustion conditions to ensure optimal and environmentally friendly combustion (Wei et al., 2021).

In addition, the composition of natural gas has a significant influence on the monetary value of the natural gas. Both, industrial and private customers of natural gas receive bills based on average values of the natural gas composition and thus the energy content. The actual energy content sometimes varies significantly from the values used for billing, which often results in financial disadvantages for the customer. Knowing the actual composition of the natural gas purchased would make it possible to create correct bills regarding the energy content (Paulus and Lemort, 2023).

The knowledge of the exact composition of natural gas is of utmost importance in many aspects. Several methods exist for determining the natural gas composition regarding the individual hydrocarbons.

Gas chromatography (GC) is considered the gold standard of natural gas analysis. The individual natural gas components are separated in a separation column and then quantitatively detected, for example, with a flame ionization detector (FID) (Rhoderick, 2003; Brown et al., 2004). The advantage of GC is the high accuracy and the high dynamic range regarding the concentrations. With a gas chromatograph, measurements with deviations of 1%–2% are possible in a dynamic range starting in the ppb range up to 100%. The flame of the FID can be problematic in places with a high risk of explosion, for example, at drilling sites. Furthermore, a variety of operating gases are required, such as helium as carrier gas, synthetic air and hydrogen as combustion gas for the FID. Additionally, the analysis time can be up to 45 min depending on the selected analysis parameters (Poole, 2021).

A number of spectroscopic methods have been tried out for natural gas analysis, which have both advantages and disadvantages compared to the established GC.

Infrared spectroscopy in various forms was tested. The wavelength range was typically 1,000–2,000 nm.

A variety of measuring instruments exist which can detect methane, the main component of natural gas, by means of infrared absorption spectroscopy (Compur Monitors, 2021; Mueller-Elektronik AG, 2023). These devices are very compact and thus allow field measurements with very short measuring times. The disadvantage of this type of measurement devices, however, is that they cannot perform a natural gas analysis and thus only provide an indication of natural gas leakage.

Infrared absorption spectroscopy can also be performed from higher altitudes. Unmanned flying objects (drones) and satellites are used for this purpose (Iwaszenko et al., 2021; Pandey et al., 2023). Their emitted laser radiation is reflected at the Earth’s surface and thus also allows conclusions to be drawn about the presence of methane in the atmosphere. The advantage of these methods is the large measurement area that can be covered. Since the reflectivity depends on the existing topography of the reflecting point and the interaction path is large due to the measurement height, cross-sensitivities causing larger deviations are to be expected with this measurement method.

Fourier transform near-infrared (FTNIR) spectroscopy is a well-known embodiment of infrared spectroscopy (Haghi et al., 2017). Based on a Michelson interferometer, an absorption spectrum is generated in conjunction with the Fourier transform, from which the proportions of the individual components of the gas sample can be determined.

Another method of infrared spectroscopy is transmission spectroscopy based on hollow-core photonic bandgap fibers (Li et al., 2012). The gas mixture to be analyzed flows into the fiber, where the interaction between the laser radiation and the natural gas occurs. By tuning the laser, an absorption spectrum can be recorded by means of an intensity detector. The resulting spectra can also be used to quantify the individual gas components.

The advantage of both methods is the comparatively short measurement time in the range of seconds compared to gas chromatography. Water exhibits strong absorption lines in the spectral range mentioned, so that large cross-sensitivities can occur, which leads to considerable measurement deviations. Furthermore, there is a temperature dependency for both methods, which negatively influences the accuracy. For these reasons, field measurements are hardly possible.

A further spectroscopy method, which enables the detection of almost all natural gas components, is Raman spectroscopy (Kiefer et al., 2008; Dąbrowski et al., 2019; Petrov et al., 2022). This measurement technique based on the Raman Effect requires laser power in the Watt range, which makes it expensive and eye safety becomes an important issue. Furthermore, a set of calibration measurements are prerequisite to obtain reliable results under a wide range of measurement conditions.

One of the most recently published spectroscopic technologies for natural gas analysis is based on proton Nuclear Magnetic Resonance (NMR) spectroscopy (Duchowny et al., 2022). The advantage of this method is the high accuracy of the results, the relative error is less than 1% in relation to the GC reference. Measurements under different temperature and pressure conditions are also possible. Nevertheless, field measurements are hardly possible since a benchtop NMR, such as the Spinsolve 60 ULTRA NMR spectrometer from Magritek (Aachen, Germany), weighs 60 kg and measures 58 × 43 × 40 cm.

Recently, a sensor based on light-induced thermoelastic spectroscopy (LITES) was presented, that is capable of detecting natural gas main components. However, the measurements were limited to synthetic gas mixtures consisting of methane, ethane and nitrogen. The maximum methane share investigated was 10% (Zifarelli et al., 2023).

Photoacoustic spectroscopy is well known as an analytical method for the detection of trace gases in the sub-ppt to ppm range (Sigrist, 1995; Wang et al., 2022). The main advantage over purely optical absorption spectroscopy is that photoacoustic spectroscopy is an offset-free technique.

When modulated laser radiation hits molecules, which absorb the energy of the photons, complex intermolecular relaxation processes can occur. Subsequently, a thermal wave is generated, which leads to a pressure wave. This phenomenon is well known as the photoacoustic effect which is applied in photoacoustic spectroscopy. The resulting sound wave is typically amplified by means of an acoustic resonator and detected by a microphone (Palzer, 2020).

QEPAS (Quartz Enhanced Photoacoustic Spectroscopy) represents a special embodiment of PAS. In this case, the microphone is replaced by a quartz tuning fork, which typically has a Q-factor greater than 10,000 (Sampaolo et al., 2022). QEPAS has already been successfully used to study the relaxation processes of gas mixtures consisting of methane, ethane and propane, each with fractions in the lower percentage range (Menduni et al., 2022). First QEPAS measurements involving the simultaneous detection of synthetic natural gas-like mixtures consisting of methane, ethane, and propane under laboratory conditions were presented (Luo et al., 2022). The evaluation is based on the signal amplitude at discrete wavelengths. The measurements are so far limited to synthetic mixtures.

To the best of our knowledge, we developed the first photoacoustic analyzer that can determine the concentrations of methane, ethane and propane of real natural gas samples. The investigated natural gas originates from the house supply of Hamburg University of Applied Sciences (Hamburg, Germany). Furthermore, we have investigated a gas sample originating from a German natural gas storage. In this regard we also present a method for preparing gas samples of atmospheric pressure for the photoacoustic measurement.

Chapter 2 provides the relevant short-chain hydrocarbons absorption spectra and describes the measurement setup and the respective measurement procedure. The third chapter presents the measurement results, which are subsequently discussed in Chapter 4.

## 2 Methods and material

### 2.1 Methane, ethane and propane absorption spectra

**FIGURE 1:**
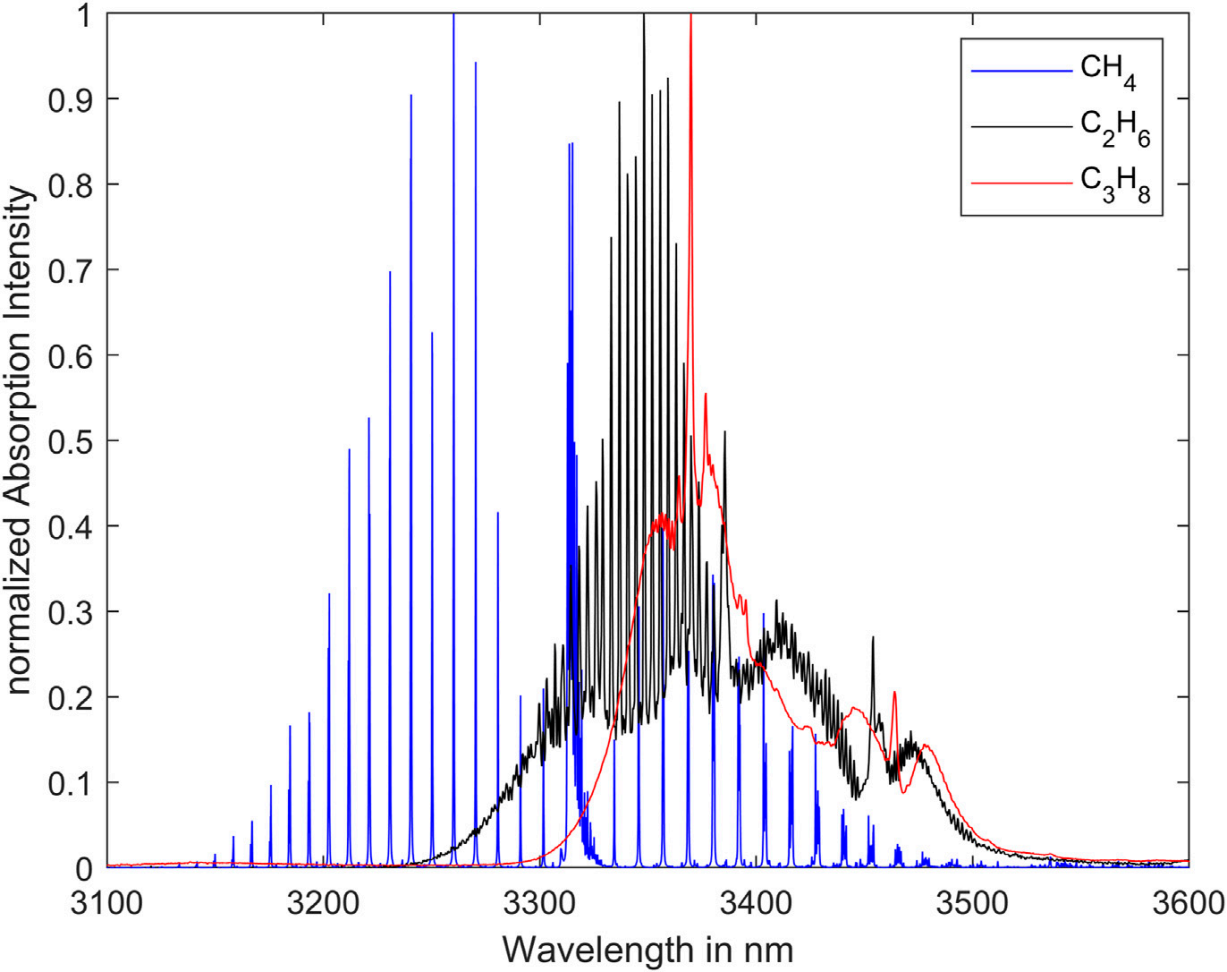
Absorption spectra of methane, ethane and propane (GEISA, 2022; Gordon et al., 2022).

**FIGURE 2:**
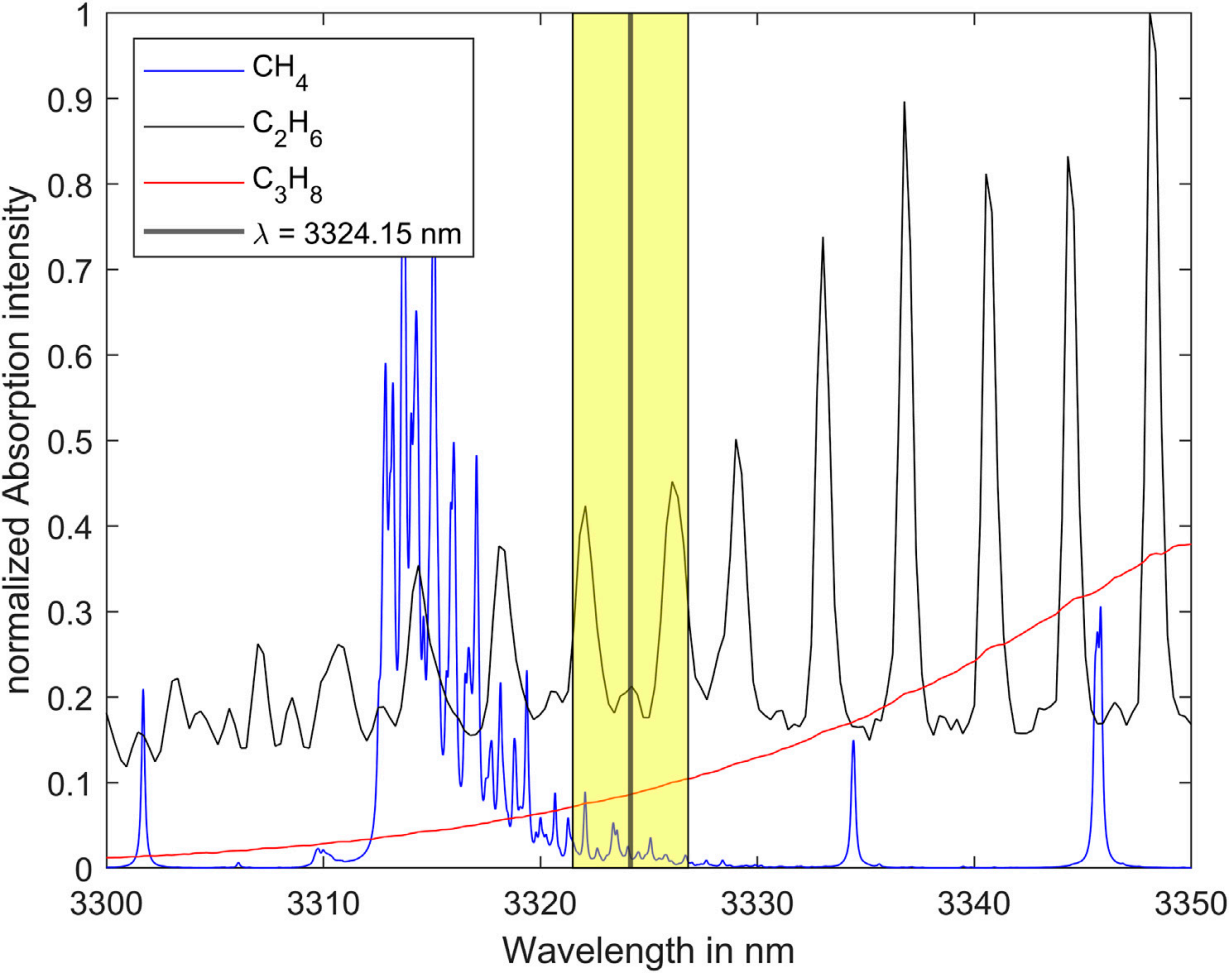
Absorption spectra of methane, ethane and propane together with the laser tuning range (yellow box) and the laser center wavelength (GEISA, 2022; Gordon et al., 2022).

The analyzer is based on an interband cascade laser (ICL) emitting in the mid-infrared range. Figure 1 shows the normalized absorption spectra of methane, ethane and propane at room temperature (296 K) and atmospheric pressure (1013.25 hPa). The absorption cross-section coefficient of methane, ethane and propane are all of a similar order of magnitude in this spectral range. The center wavelength of the laser has been empirically selected to be 3324.15 nm and is displayed by the vertical dark line in the yellow box in Figure 2. To clearly identify several components of a gas mixture, it is necessary to analyze the spectrum in a specific range. For this purpose, the laser is spectrally tuned. The yellow box represents the laser tuning range whose spectral bandwidth is approximately 5.3 nm.

### 2.2 Measurement setup

**FIGURE 3:**
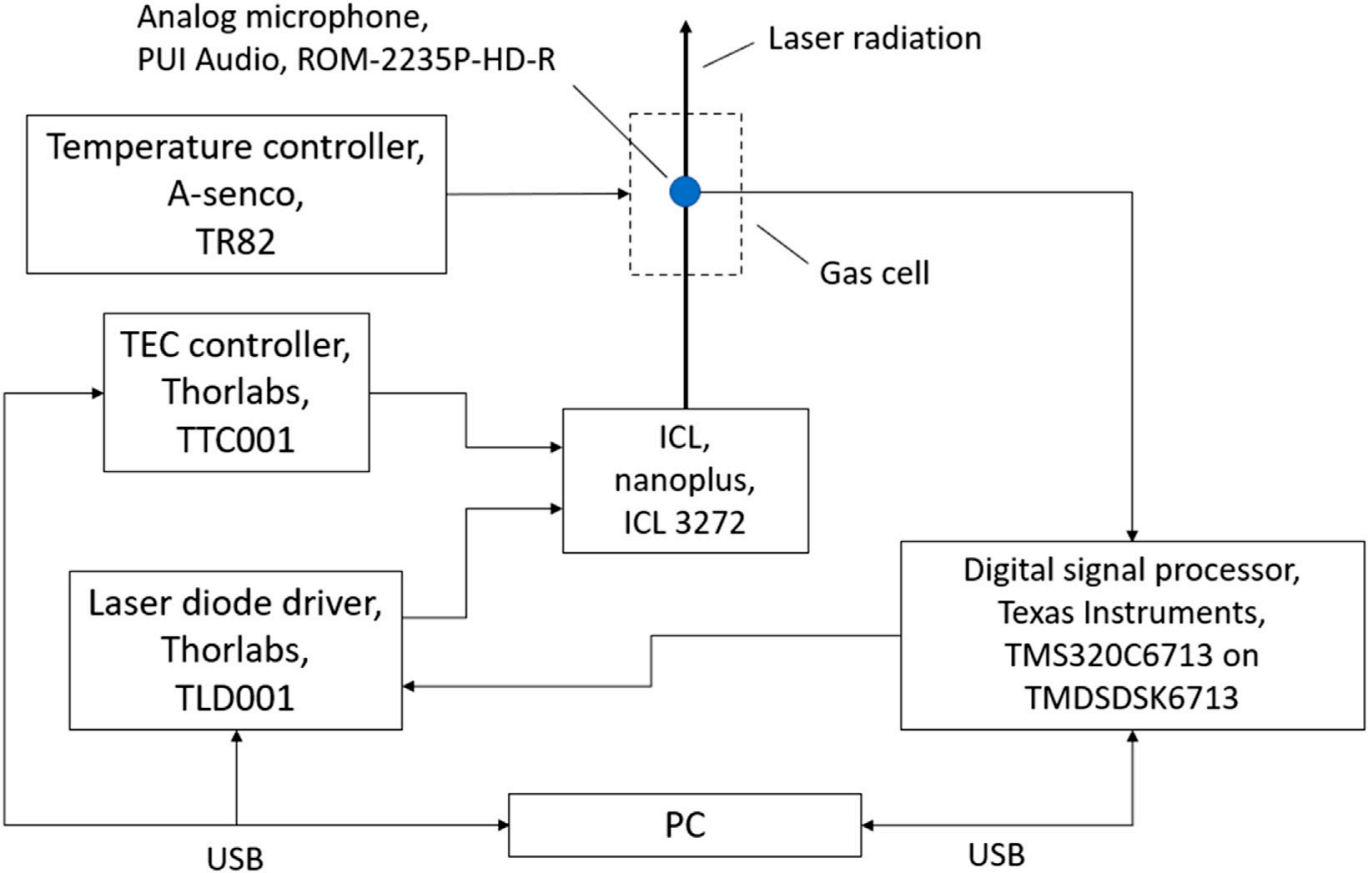
Experimental setup.

The measurement setup is schematically shown in Figure 3. The ICL 3272 manufactured by nanoplus GmbH (Gerbrunn, Germany) emits radiation around 3323 nm with a spectral linewidth smaller than 20 MHz. The laser chip temperature can be varied between 20 and 30°C. Laser operating currents between 19 and 120 mA allow stimulated emission. For the operation of the ICL the Thorlabs (Newton, MA/USA) laser diode driver TLD001 and the temperature controller TTC001 are used. The TLD001 allows to set the laser current with an accuracy of 10 µA with a noise level below 3 µA rms. Installed in a TO66 housing, the ICL chip temperature is controlled using a temperature sensor and a Peltier element. The single-mode emission can be continuously tuned between 3318.41 nm and 3330.64 nm. Approximately 25 mW output power can be reached at maximum. The cylindrically symmetrical photoacoustic measurement cell, that the laser beam centrally passes, is designed according to the established H geometry boasting a longitudinal resonance around 3 kHz (Nodov, 1978). The photoacoustic signal is detected with the analog microphone ROM-2235P-HD-R from PUI Audio (Fairborn, OH/USA) which is mounted in the center of the cell, membrane flush with the wall. The microphone exhibits a diameter of 5.8 mm and a detection sensitivity of −35 ± 3 dB at 1 kHz and 50 cm distance. The temperature of the measurement cell is controlled by the A-senco (Aalen, Germany) temperature controller, which heats the cell to a constant temperature above the ambient temperature by means of two heating mats. The maximum heating power is 30 W. The demodulation is performed on a Texas Instruments (Dallas, TX/USA) TMS320C6713 digital signal processor (DSP) which is mounted on the Texas Instruments TMDSDSK6713 evaluation board. The laser current modulation is controlled by the digital signal processor as well. A PC is used to control laser diode driver, TEC controller and DSP. The photoacoustic signals calculated by the DSP are recorded by the PC as function of the average current. These spectra of known mixtures serve as basis for a PLSR model based on a MATLAB script, which can subsequently be used to calculate the concentrations of the single components of unknown mixtures.

**FIGURE 4:**
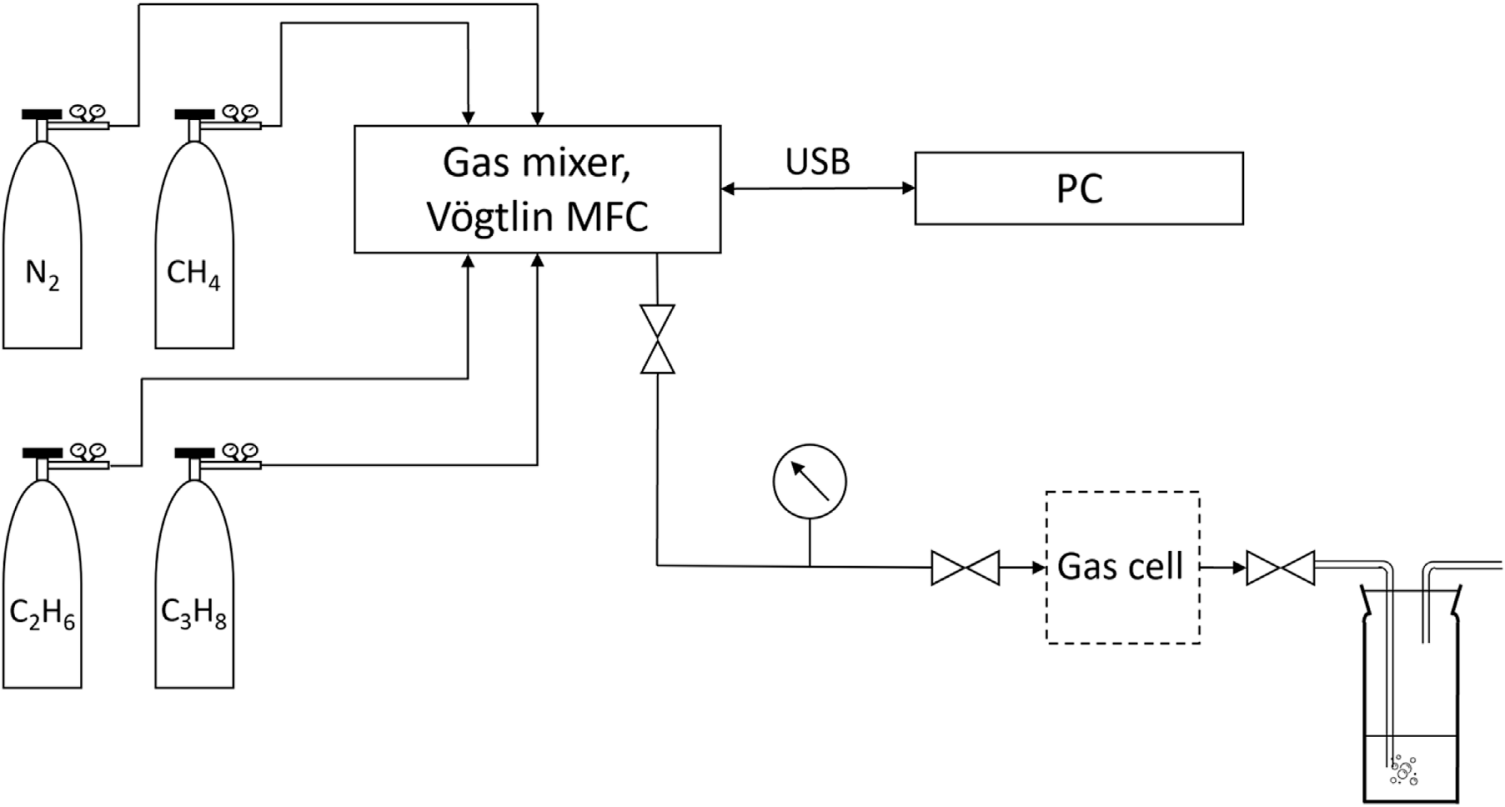
Gas flow system.

The gas flow system for the generation of the calibration mixtures is schematically shown in Figure 4. The gas is provided in four containers:

- CH_4_: 10 L geometric volume, 200 bar filling pressure, purity: 2.5, distributor: Westfalen AG,
- C_2_H_6_: 1 L geometric volume, 12 bar filling pressure, purity: 2.0, distributor: Westfalen AG,
- C_3_H_8_: 1 L geometric volume, 7.3 bar filling pressure, purity: 2.5, distributor: Westfalen AG,
- N_2_: 10 L geometric volume, 200 bar filling pressure, purity: 5.0, distributor: Westfalen AG.

All gas mixtures for calibration are generated using the 4-channel gas mixer based on Vögtlin Instruments GmbH (Muttenz, Swiss) mass flow controllers (MFC), which are distributed by HTK Hamburg GmbH (Hamburg, Germany). The maximum relative error of a single MFC is specified to be up to 1%.

The validation measurements of the natural gas samples were carried out using the Shimadzu (Kyōto, Japan) GC 2014, which is equipped with an FID and a thermal conductivity detector (TCD). The relative measurement error is specified to be up to 2%.

**FIGURE 5:**
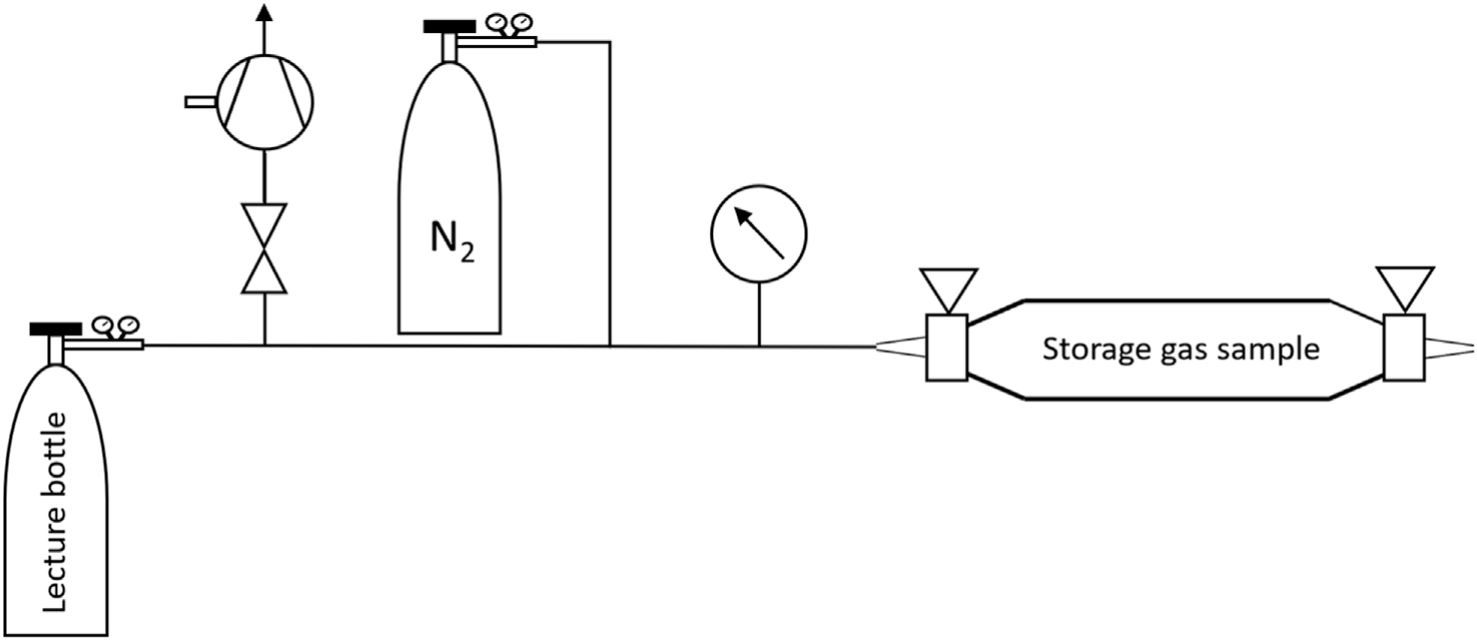
Storage gas preparation system.

The storage gas sample was provided by GEO-data (Garbsen, Germany) in a gas collection tube with a geometric volume of about 350 mL. The storage gas originates from a natural gas storage facility within Germany. Its preparation has been performed using the setup shown in Figure 5. A lecture bottle (geometric volume approximately 400 mL) was evacuated by the VACUUBRAND GmbH and Co. KG (Wertheim, Germany) Chemistry-HYBRID pump down to 0.1 mbar absolute pressure.

The natural gas samples used are from Hamburger Energiewerke GmbH (Hamburg, Germany) and were obtained from the house gas connection of the Hamburg University of Applied Sciences.

### 2.3 Measurement procedure

At a constant temperature of 20°C, the ICL applied in this investigation is operated in the injection-current modulation mode. The injection-current consists of two parts. One part is continuously tuned between 66.00 and 120.12 mA covering a spectral range of approximately 5.3 nm. The other part is sinusoidally modulated with an amplitude of 1.18 mA which corresponds to a spectral range of approximately 0.1 nm.

The temperature of the measurement cell is set and controlled to constant 30°C by the temperature controller.

The microphone signal is sampled at a sampling rate of 44.1 kHz. The recorded spectra consist of 315 points, with each spectral component calculated from 1,500 samples. The demodulation of the microphone signal on the DSP, based on the Goertzel algorithm, is performed at the injection-current modulation frequency at 3,528 Hz (Engelberg, 2008). The resulting spectra correspond approximately to the first derivative of the absorption spectra, which emphasizes the changes in the absorption coefficient, induced by changes in the single gas concentrations.

**FIGURE 6:**
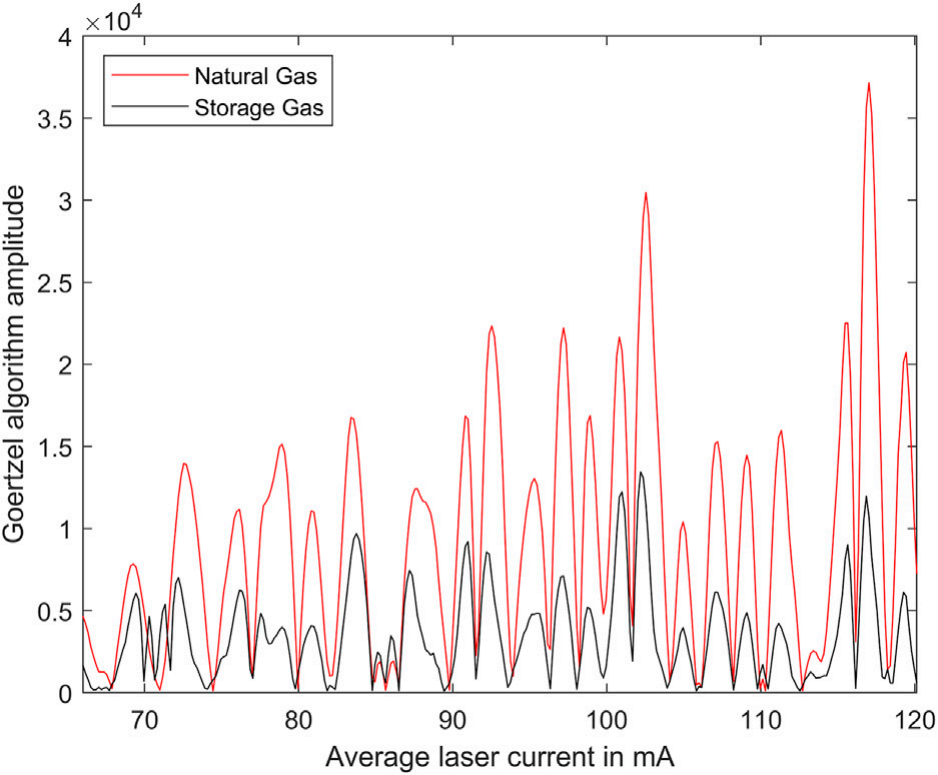
Exemplary photoacoustic spectra of natural gas and storage gas measured on 28 June 2023 and 29 June 2023, respectively.

Figure 6 shows exemplary photoacoustic spectra, i.e., the PA signals as function of the average laser current after the Goertzel algorithm based demodulation on the DSP. All measurements were taken at a sample (cell) temperature of 30°C and a pressure of 1,013 hPa.

The investigation of complex gas mixtures such as natural gas requires a multivariate approach to determine the methane, ethane and propane shares. As shown in previous investigations, Partial Least Squares Regression (PLSR) proved to be suitable for this task (Menduni et al., 2022). Reference calibration measurements of the photoacoustic sensor are required to predict the individual shares of the natural gas samples with a high precision and reliability. A training data set of 20 different mixtures has been used, all concentrations are uniform distributed. All calibration gas mixtures consist of methane, ethane and propane. The calculation of the PLSR model is performed on the PC. The magnitudes of the spectrum represent the predictor scores and the corresponding known concentrations provide the response scores. The beta-parameters are the result of the PLSR model calculation, which are the basis for the prediction of gas mixture compositions. By multiplication and summation operations of the beta-parameters with the spectra of unknown mixtures, the concentrations of its hydrocarbon components are calculated (Rosipal and Krämer, 2006).

A complete measurement run including calculation of the unknown hydrocarbon concentrations of a gas mixture requires approximately 12 s.

The filling and purging of the measuring cell are carried out as shown in Figure 4 using gas samples with overpressure relative to the atmosphere. If the gas samples do not have an overpressure, they must be prepared according to Figure 5. In this case, a lecture bottle is evacuated. The pressure in the system is less than 0.5 mbar absolute pressure when evacuated. The valve of the gas collection tube is subsequently opened and a constant pressure lower than the ambient pressure is established in the system. To generate the necessary overpressure relative to the atmosphere, the lecture bottle is then filled with nitrogen up to 4 bar above atmospheric pressure. This procedure allows a sample preparation adequate for the measurement cell. The concentration ratios of the single hydrocarbons, except for nitrogen, remain unchanged.

For GC analysis, the samples are injected into the GC by means of a dosing loop which, in conjunction with a valve circuit, dispenses the analyte onto the packed column. After 12 min retention time the measuring results of the TCD and the FID are available. The evaluation of the chromatograms is based on calibration curves, which were measured using the following calibration gases:

- Methane: 2.5%, 50%, 100%
- Ethane: 0.85%, 1%, 100%
- Propane: 0.85%, 100%
- Nitrogen: 100%

The residual gases are in all cases nitrogen.

## 3 Results

Table 1 lists all gas samples that were investigated. The natural gas samples of the supplier Hamburger Energiewerke GmbH have been investigated at three different times. Due to the ambient pressure of the storage gas sample, it had to be prepared and diluted as described in Section 2.3 using the setup shown in Figure 5. PAS did not detect any propane in this sample and the GC result was “less than 1‰“.

**TABLE 1:** Analysis results.

Sample type	Date	PAS			GC		
		Methane (%)	Ethane (%)	Propane (%)	Methane (%)	Ethane (%)	Propane (%)
Natural gas	26 May 2023	86.11	4.98	0.92	85.23	4.70	0.96
	28 June 2023	85.03	4.95	0.67	85.10	4.96	0.87
	2 August 2023	82.35	4.60	0.62	84.33	4.41	0.77
Storage gas	29 June 2023	6.79	0.41	-	6.78	0.48	<0.1

**FIGURE 7:**
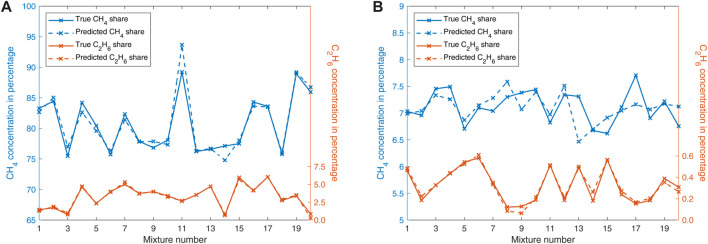
**(A)** Results of the leave-one-out cross-validation of natural gas (propane is hidden for clarity). **(B)** Results of the leave-one-out cross-validation of storage gas.

Figures 7A, B show the true and the predicted shares for methane and ethane exemplarily. The absolute root mean square errors (RMSE) for the methane, ethane and propane concentrations in Figure 7A are 1.34%, 0.19% and 0.13%, respectively. The absolute RMSE for methane and ethane concentrations in Figure 7B are 0.29% and 0.03%, respectively.

**FIGURE 8:**
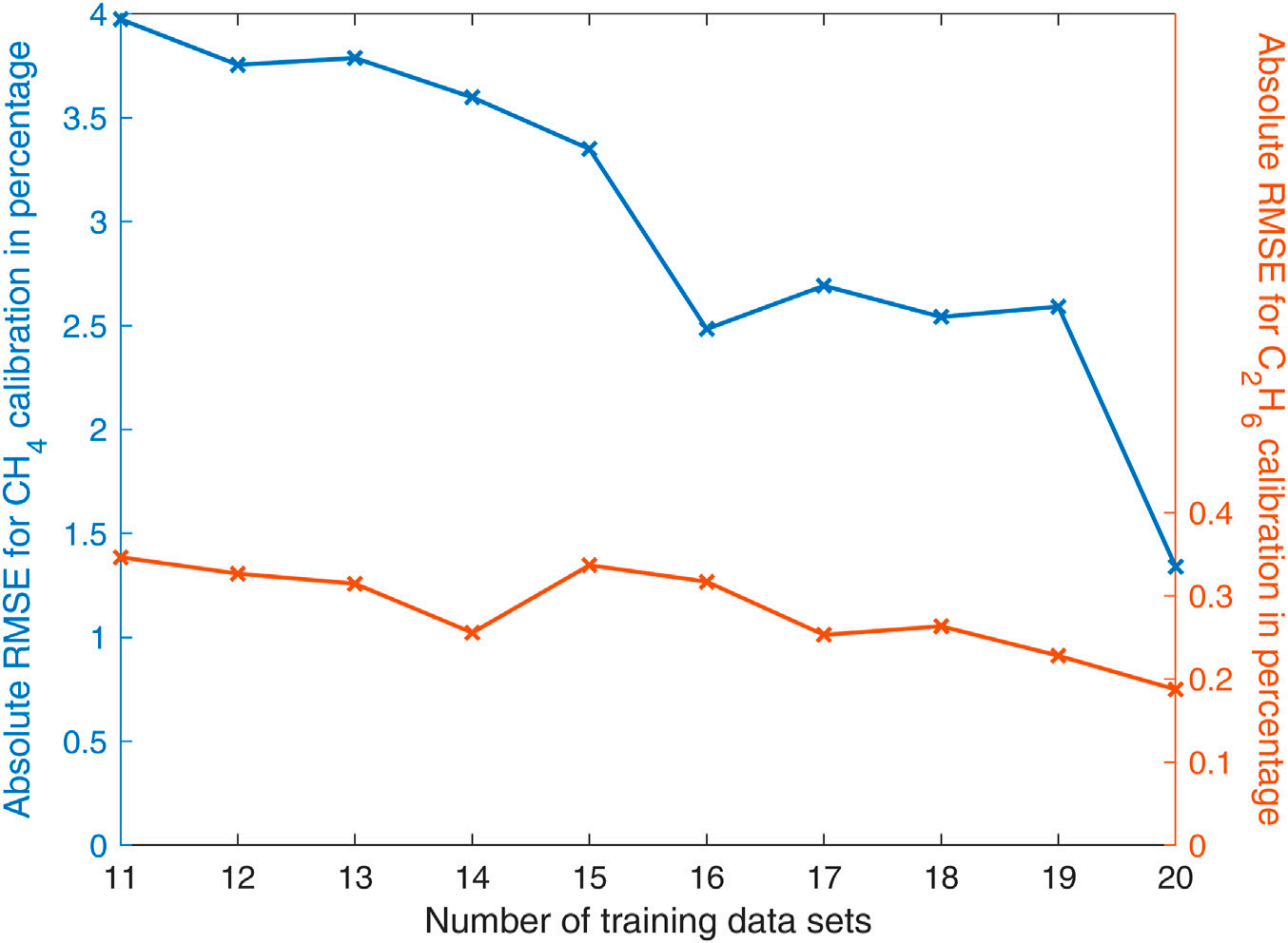
Absolute RMSE as function of the number of training data sets.

The number of training data sets significantly influences the absolute RMSE. Figure 8 illustrates this relationship.

## 4 Discussion

Figure 6 shows an exemplary photoacoustic spectrum. It does not perfectly correspond to the derivative of the absorption spectrum, due to the nonlinear spectral behavior of the injection-current modulation and the missing normalization regarding the laser output power.

As shown in Tab. 1, the PAS results are in good agreement with the GC results. If we consider that the GC exhibits a relative error of up to 2%, the agreement can be described as excellent.

As displayed in Figure 8, an increase of the number of training data sets leads to a significantly decreasing absolute RMSE. Notwithstanding only a relatively low number of 20 training data sets were used for this investigation, accurate results could be achieved. A further increase of the number of training data sets would further decrease the error.

The natural gas analysis on 28 June 2023 delivered a methane concentration of 85.03% (PAS) and 85.10% (GC), respectively (see Tab. 1). Both values do not agree with the 91.95% officially published by Gasnetz Hamburg GmbH (Hamburg, Germany) for this month (Gasnetz Hamburg GmbH, 2023). The true value is almost 7% smaller than the value used for invoicing.

In conclusion it can be stated that, photoacoustic spectroscopy in combination with PLSR is a suitable method for analyzing natural gas samples as well as storage gas samples in a wide dynamic range. Due to the short analysis time of approximately 12 s and the absence of any operating gases, the presented PAS-based sensor could be further developed into an alternative to GC that is attractive in terms of price and performance.

## Data Availability

The original contributions presented in the study are included in the article/Supplementary material, further inquiries can be directed to the corresponding author.
